# Experimental implantation of an arterial substitute made of silicone reinforced with polyester fabric in rabbits

**DOI:** 10.6061/clinics/2017(12)10

**Published:** 2017-12

**Authors:** Laila Massad Ribas, Inez Ohashi Torres, Fernanda Appolonio, Karina Paula Domingos Rosa, Fabio Rodrigues Ferreira do Espírito-Santo, Nelson De Luccia

**Affiliations:** Departamento de Cirurgia, Faculdade de Medicina FMUSP, Universidade de Sao Paulo, Sao Paulo, SP, BR

**Keywords:** Vascular Grafting, Polydimethylsiloxane, Polyesters, Silicon, Aorta, Blood Vessel Prosthesis

## Abstract

**OBJECTIVES:**

The aim of this study was to analyze silicone tubes with an internal diameter of 4 mm as a possible material for vascular prostheses.

**METHODS:**

Grafts were implanted into the infrarenal aortas of 33 rabbits. Fluoroscopic examinations were performed within 150 days after surgical implantation. Sample grafts were analyzed via electron microscopy to evaluate the eventual endothelialization of the prostheses.

**RESULTS:**

The patency rates of the prostheses were 87% (±6.7%) after 30 days, 73% (±9.3%) after 60 days and 48% (±12%) after 120 days. The material presented characteristics that support surgical implantation: good tolerance promoted by polyester tear reinforcement, ease of postoperative removal and a lack of pseudoaneurysms. However, intimal hyperplasia was a limiting factor for the patency rate.

**CONCLUSIONS:**

We concluded that polydimethylsiloxane has limited potential as an alternative material for small vascular prostheses.

## INTRODUCTION

Graft replacement of large vessels, such as the aorta and the iliac and femoral arteries, is reasonably achieved using currently available synthetic tubes, such as those composed of polytetrafluoroethylene (PTFE) and Dacron 1-5. However, in small vessels, none of these materials have proved to be superior or equal to the saphenous vein 6-10, which is regarded as the conduit of choice for peripheral revascularization 11-14.

A challenge that can arise is the lack of an available vein for arterial replacement. A substantial amount of research has been performed in this field, and there are several options for solving this problem. Cryopreserved donor veins 15,16 and biosynthetic materials are examples of alternatives described in vascular surgery studies 17,18. However, the search for an effective alternative for the replacement of small vessels remains open 19,20.

Polydimethylsiloxane (PDMS), or silicone, has been used in medicine since the 1960s 21,22. Due to its characteristics, silicone has become one of the most frequently used materials for prosthetic replacement in various contexts, such as breast reconstruction 23. The use of PDMS in the form of various types of catheters for the intravenous administration of substances is also widespread and universally accepted 24,25.

The characteristics that make this material attractive for these uses are its excellent thermal stability, good wetting properties, physiological inertness, excellent long-term biostability and low thrombogenicity 26-28.

Given that the search for an ideal vascular prosthesis continues and that silicone is a material that promotes few tissue reactions, this study aims to propose an experimental model and test the hypothesis that PDMS is a suitable substitute for small vessels.

## MATERIALS AND METHODS

All surgical procedures were performed in domestic rabbits (*Oryctolagus cuniculus)* in the Department of Surgery in the Faculty of Medicine of the University of São Paulo. The animals were provided by that institution’s animal house and were cared for in accordance with principles established by the Animal Welfare Act and the NIH Guide for Care and Use of Laboratory Animals.

This study was conducted with the approval of the ethics committee of the Faculty of Medicine of the University of São Paulo.

A tubular prosthesis made ??of PDMS (silicone) reinforced with polyester fabric was implanted into the infrarenal aorta of each animal.

### Synthetic prosthesis

**Figure 1:**
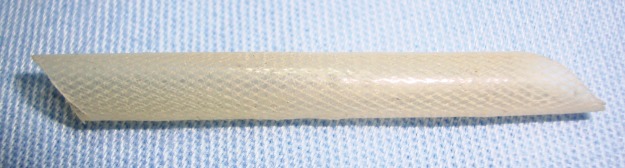
PDMS prosthesis with a tubular wall of 0.4 mm and an internal diameter of 4 mm.

The prostheses were prepared in accordance with a patent-registered design 29. Medical-grade silicone in liquid form was mixed with a curing agent and applied over a metal mandrel covered with a polyester fabric to increase the silicone’s tear resistance. To achieve uniform curing, rotational movement of the mandrel was maintained, initially at room temperature and then in an oven to achieve a post-cure temperature of 110°C for 30 minutes. The PDMS prosthesis exhibited a tubular wall of 0.4 mm and an internal diameter of 4 mm (Figure 1). Despite attempts to create a porous tube, the final product was nonporous, and the internal and external surfaces were composed of silicone. The fabric material remained inside the wall simply to provide reinforcement.

### Anesthetic procedures

Animals were anesthetized with a mixture of ketamine hydrochloride (35 mg/kg i.m. Ketalar 10%; Cristalia, São Paulo, Brazil) and xylazine hydrochloride (5 mg/kg i.m. Rompum 2%; Bayer AG, Leverkusen, Germany). During the surgical procedures, the animals received saline solution (0.9% sodium chloride) through a 22G catheter cannulated in the marginal ear vein.

### Surgical technique

An anterior laparotomy was performed, and a transperitoneal approach allowed for the dissection of 3 to 4 cm of the infrarenal aorta. A specifically developed self-static retractor was used to avoid evisceration during this exposure. The lumbar arteries were carefully preserved. Before the aorta was clamped with microsurgical clamps, sodium heparin (200 U/kg i.v.; Hepamax, Blausiegel, São Paulo, Brazil) was administered.

Anastomoses of the PDMS in the aorta were completed using an end-to-side technique and a continuous 7-0 polypropylene suture. At the end of the proximal anastomosis, blood flow was released for a few minutes for ischemic conditioning.

At the end of the vascular anastomosis, pulses were checked, and the aorta was ligated with 4-0 cotton and cut between the anastomotic sites.

The animals were evaluated for up to 150 days. All surviving animals were subjected to fluoroscopic examination via retrograde femoral contrast injection.

### Prosthesis evaluations

The prostheses were evaluated using aortic fluoroscopy. Images were acquired on a Diasonics OEC^®^ 9000 fluoroscopy scanner (Salt Lake City, Utah, USA). Dissection of the femoral artery was performed via unilateral inguinal incision to perfuse the contrast agent diatrizoate meglumine (Reliev 60%; BerliMed S.A., São Paulo, Brazil).

At the end of the experimental procedures, the animals were euthanized while they remained anesthetized with 19.1% potassium chloride (Isofarma, Eusébio, CE, Brazil), and their bodies were disposed of in accordance with the surgical department’s routine procedures.

The prostheses were removed after euthanasia, and samples were sent for scanning electron microscopy; images were obtained using a Philips XL30 system (FEI, Hillsboro, Oregon, USA).

### Statistical analysis

The Kaplan-Meier method was used to analyze risk of occlusion and patent condition. Statistical analyses were performed using SPSS 18.0 software.

## RESULTS

Surgical procedures were performed in 64 animals. Thirty animals (46.9%) survived until late evaluations. Early mortality was observed in 23 animals (35.9%). Eleven animals (17.2%) developed paraplegia in the immediate postoperative period, and these animals were not considered for long-term follow-up.

All 30 surviving animals were submitted to angiographic control at the end of the observational period. In three paraplegic animals that were not considered for late follow-up, angiography was performed to diagnose the status of the graft. Another three animals were excluded from late follow-up because early occlusion (at less than 7 days) of the graft was detected. The remaining 27 animals were analyzed using Kaplan-Meier survival/patency curves.

### Surgical characteristics

The walls of the prostheses had characteristics of flexibility and hardness that allowed for easy passage of the needle and retention and containment of the suture lines. Thus, satisfactory hemostasis was achieved at the end of the experiments, and the presence of a pulse distal to the anastomoses attested to the immediate patency of the prostheses in all animals.

### Prosthesis evaluations

**Figure 2:**
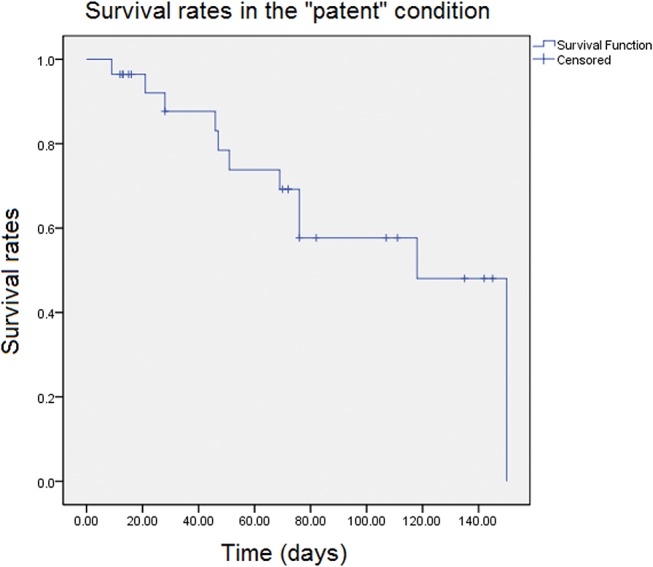
Proportion of "survivors" in the patent condition. The patency rates of the prosthesis were 87% (±6.7%) after thirty days, 73% (±9.3%) after sixty days, 57% (±11%) after ninety days and 48% (±12%) after one hundred twenty days.

**Figure 3:**
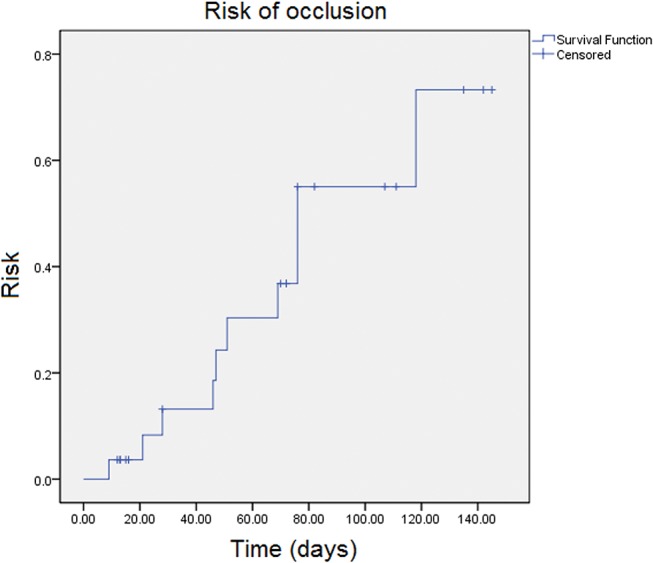
Risk of occlusion of the prosthesis.

The patency rates of the prosthesis were 87% (±6.7%) after thirty days, 73% (±9.3%) after sixty days, 57% (±11%) after ninety days and 48% (±12%) after one hundred twenty days (Figure 2). The risk of occlusion is indicated in Figure 3.

### Macroscopic analysis

Peri-implant fibrous tissue was easy to identify, and a cleavage plane between the periprosthetic tissue reaction and the tube was observed. Neither aneurysmal dilatation of the implant nor pseudoaneurysm formation in the suture lines was observed. Inside the occluded grafts, whitish thrombi vwere observed that were likely caused by intimal hyperplasia, which was subsequently confirmed via scanning electron microscopy.

### Scanning electron microscopy

**Figure 4:**
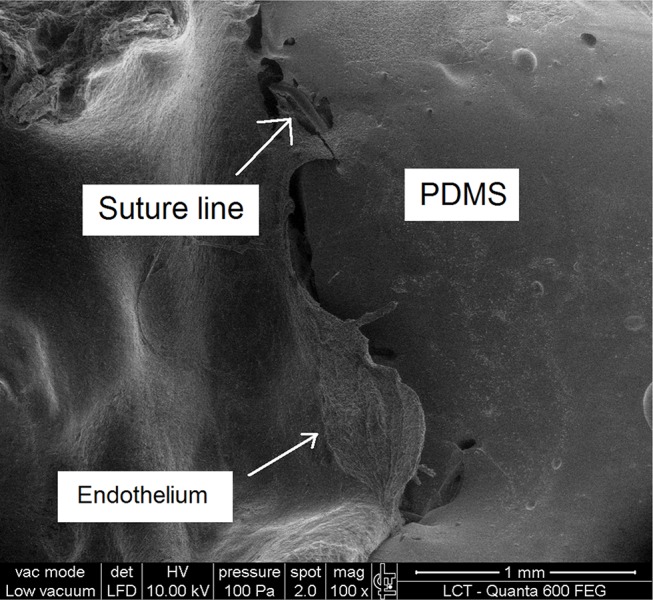
Electron microscopy image showing endothelial growth from the native vessel toward the PDMS graft, which is consistent with intimal hyperplasia.

Electron microscopy was used to provide additional information in this study. It was possible to observe endothelial growth from the native vessel toward the PDMS graft, a finding consistent with intimal hyperplasia (Figure 4).

## DISCUSSION

Vascular prostheses have been used for several decades to restore blood flow at many sites. However, although the functions of such prostheses have been well established for large vessels, the use of these prostheses is greatly limited in vessels with a diameter of less than four millimeters 9.

This study demonstrated that the patency rate for PDMS prostheses after ninety days was 57% (±11%). Nordestgaard et al. 30 performed a similar study but used ePTFE prostheses with a diameter of three millimeters that were also implanted into rabbit aortas. Those authors reported a patency rate of 82% after ninety days. In 2012, Zheng et al. 31 tested polycaprolactone prostheses coated with arginine-glycine-aspartic acid in the carotid arteries of ten rabbits, and the patency rate after four weeks was 100%. However, the authors discussed their short observation period and acknowledged that one month is not sufficient for vascular regeneration. If we considered only thirty days of follow-up, then we would have observed 87% patency (±6.7%) in our experiments.

Intimal hyperplasia is the result of natural cicatrization after vascular injuries, especially in anastomotic regions, and is a major cause of vascular stenosis and occlusions 32. Anastomotic intimal hyperplasia is more likely to develop for synthetic prostheses than for vein grafts. Given that the endothelium is responsible for preservation of the intravascular structure 33,34, it is natural to search for graft endothelialization. One method for achieving endothelial growth relates to graft porosity. The porosity concept for vascular grafts and the growth of endothelial cells that could pave the inner surface of such grafts remain controversial in medical science.

Theoretically, pores are scaffolds for the passage and habitation of vascular cells through the graft lumen. Thus, it was thought that larger spaces between graft nodes would correspond to greater cell housing 35. However, intermediate porosity is considered to be more suitable for preventing intimal hyperplasia 36. In contrast, Lumsden et al. 37 achieved satisfactory results from coating PTFE prostheses with non-porous silicone to promote a smooth and uniform surface and limit internal tissue growth beyond the transanastomotic region. Based on that study, we developed an experimental model to test the hypothesis that non-porous silicone could be a suitable prosthesis material for small vessels. We believe that our patency results are consistent with the development of intimal hyperplasia in the anastomotic region. Electron microscopy generated images compatible with endothelial growth from the anastomotic region. Further studies with porous silicone could demonstrate the difference between porous and non-porous grafts and elucidate the cause of intimal cell growth in synthetic prostheses.

In evaluations of prostheses, patency has always been demonstrated via contrast injection into the aorta (using arteriography performed via retrograde catheterization) and long-term monitoring. Occlusions that occur in less than thirty days are related to surgical failures, such as technical failures with respect to anastomosis, prosthesis placement, prosthesis folding or insufficient blood flow 38. In our study, we performed prosthesis evaluation for up to 150 days. Within this period, we aimed to perform arteriography as late as possible.

The use of polyester as reinforcement for the PDMS was intended to prevent tears during prosthesis suturing because PDMS has a low tear tolerance. This coating appeared to be useful because these events did not occur during any surgical procedure. Moreover, pseudoaneurysms were not observed.

Although the silicone graft did not show satisfactory results with respect to long-term patency, the use of this material in other forms, such as sheets, has already been tested. An experimental study that used the same animal model produced extremely good results for patches in the aortas of rabbits 39.

The tested material exhibited characteristics that support surgical implantation: good tolerance promoted by polyester tear reinforcement, ease of postoperative removal and lack of pseudoaneurysms. However, intimal hyperplasia was a limiting factor for the patency rate. We concluded that PDMS has limited potential as an alternative small vascular prosthesis material.

## AUTHOR CONTRIBUTIONS

All of the co-authors participated in the surgical procedures. De Luccia N also contributed to the statistical analysis and supervised the entire study.
